# Misleading Mechanical Falls in an Elderly Female Patient With Hypervirulent Klebsiella pneumonia Infection

**DOI:** 10.7759/cureus.96891

**Published:** 2025-11-15

**Authors:** Yun Hung Chor, Mustafa Mohammed

**Affiliations:** 1 Emergency Department, Heartlands Hospital, University Hospitals Birmingham NHS Foundation Trust, Birmingham, GBR

**Keywords:** clinical case report, disseminated infections, elderly falls, elderly trauma, emphysematous osteomyelitis, geriatric medicine, hypervirulent klebsiella pneumoniae, klebsiella pneumonea, mechanical fall, multidisciplinary approach

## Abstract

Falls in elderly patients are a common presentation in the emergency department and are commonly labelled as “mechanical” falls. We present the case of an unusual fall in an elderly patient who initially attributed her pain in the hip to the fall. Patient presented again with presumed mechanical falls and hip pain. She was later found to have disseminated infection with emphysematous osteomyelitis of the pelvis and neck abscess due to hypervirulent *Klebsiella pneumonia *(hvKp). She was managed conservatively without surgical intervention with a prolonged course of antibiotics, leading to a successful recovery. This case highlights the disseminated potential of hvKp beyond the classic liver abscess, such as emphysematous osteomyelitis. It also serves as an important reminder that a "mechanical fall" can be a misleading diagnosis. A fall may be the first sign of a systemic illness. Therefore, a thorough history should be taken, and a detailed examination should be conducted to manage such cases properly.

## Introduction

Falls in elderly patients are a common presentation in the emergency department and can be an initial signal of underlying illness or health decline. The term "mechanical fall" is unclear, wrongly used, and does not help inform patient outcomes. Therefore, a thorough history should be taken, and a detailed examination should be conducted to manage such cases properly [1]. Hypervirulent *Klebsiella pneumoniae* (hvKp) was initially discovered in Asia and is now spreading globally. HvKp strains possess specific virulence factors, such as K1/K2 serotypes, rmpA/rmpA2 for hypercapsule production and high-efficiency siderophores like aerobactin, which enable them to evade the immune system and metastasize to various organs [2]. It has been reported that hvKp can disseminate to other organs such as the liver and the brain. Unlike classical *K. pneumoniae*, it can affect even immunocompetent individuals [3].

We present a case of an unusual fall in an elderly patient who thought that her hip pain was due to the fall. However, the patient was found to have an osteomyelitis due to *K. pneumoniae*, which could have been missed in her earlier presentation. This case also highlights the disseminated potential of hvKp beyond the classic liver abscess, such as emphysematous osteomyelitis, which was initially masked by a presumed mechanical fall.

## Case presentation

A female patient in her late 60s with a past medical history of type 2 diabetes mellitus presented to the hospital with right hip pain after falling down four flights of stairs onto her buttock. Vital signs were within normal range. An X-ray was performed, which showed no evidence of fracture. No blood tests were performed, as the patient reported that the fall was purely mechanical. Therefore, the patient was discharged home with safety netting advice after assessment by the physiotherapy team, who were satisfied with her mobility as the patient was walking independently with no aids.

Three days later, the patient re-presented to the hospital in the resuscitation room of the emergency department (ED) as a geriatric major trauma after another fall; this time, the mechanism of the fall was from the top of a staircase to the bottom. At this point, further history was obtained in which the patient reported having hip pain before even the first fall. She had a recent travel history of returning from a pilgrimage to Mecca one month ago.

**Figure 1:**
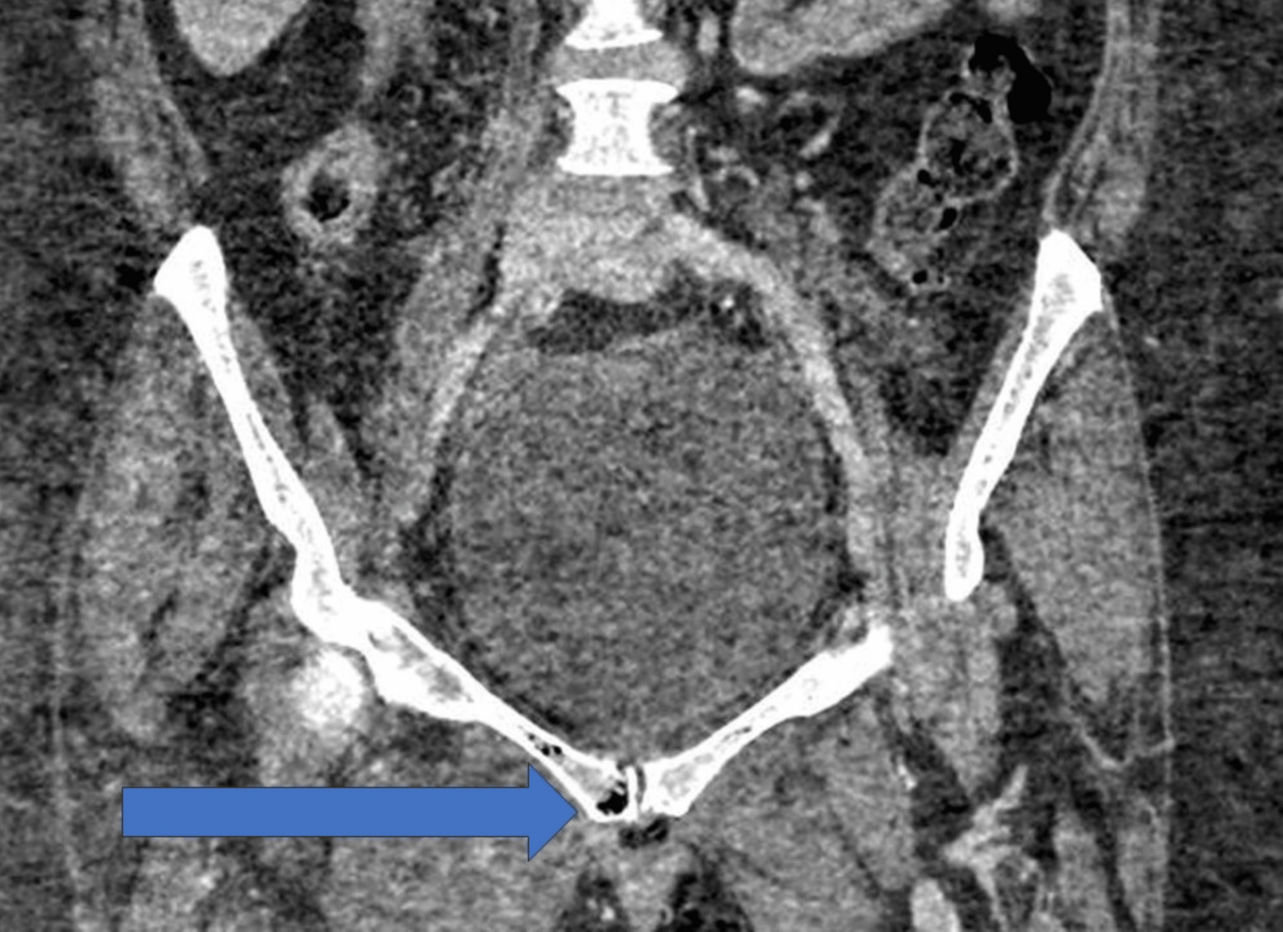
Axial CT image of the pelvis showing air in the pubic bones (emphysematous osteomyelitis) at admission Emphysematous osteomyelitis seen with intraosseous and intramuscular gas surrounding the pubic symphysis, consistent with gas‑forming infection.

She was afebrile and remained haemodynamically stable. Due to the significant mechanism of injury, computed tomography (CT) scans of the head, neck, thorax, abdomen, and pelvis were performed. CT imaging revealed scalp hematomas and a grade II liver laceration in segment VII without associated perihepatic hematoma or active bleeding. An incidental non-occlusive thrombus was noted within the inferior vena cava (IVC) without features of traumatic vascular injury. Abnormal appearances of the pubic bones were observed without evidence of fracture, consistent with emphysematous osteomyelitis accompanied by inflammatory phlegmon and intraosseous as well as intramuscular air (Figure 1). As hip pain preceded the first fall, it suggested the infection was the primary cause, not the consequence, of the trauma. Initial blood tests showed raised C-reactive protein (CRP) of 295 mg/L, white blood cell (WBC) of 29.52 10⁹/L and HbA1c of 73 mmol/mol.

**Figure 2:**
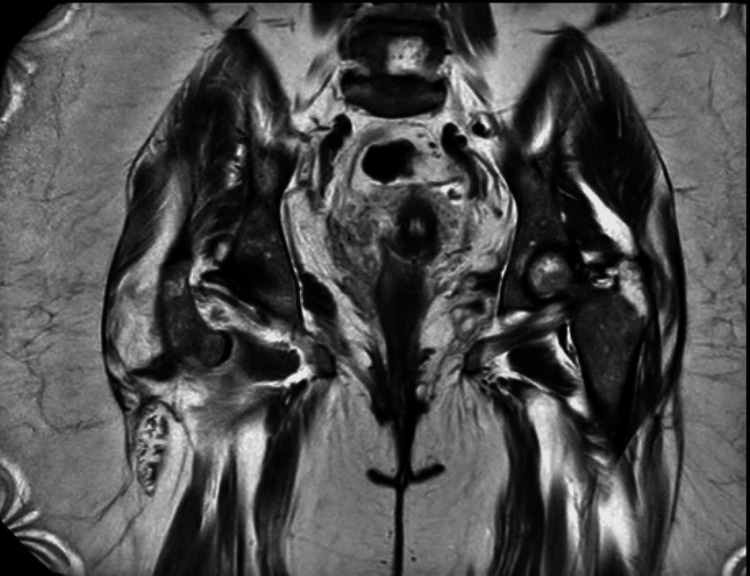
MRI (coronal view) of the pelvis showing extensive soft tissue oedema and swelling centred at pubic symphysis and extending along bilateral adductor muscles, lateral to greater trochanter and along the iliacus muscle within the pelvis (emphysematous cellulitis/fasciitis) There was no drainable abscess.

Given the complexity of the case, multiple specialties were involved, and the ED team empirically started intravenous (IV) meropenem and IV clindamycin to cover for suspected necrotising fasciitis. General Surgery and Trauma & Orthopaedics (T&O) were referred to rule out necrotising fasciitis. Magnetic resonance imaging (MRI) was planned, and they did not feel that surgical intervention was indicated, as there was no drainable collection on the CT scan. The T&O team also performed an inspection of the perineum, which showed no signs of inflammation. There was tenderness over the right hip region; however, no skin changes or swelling were seen. The subsequent MRI, as advised by T&O, showed extensive soft tissue oedema and swelling centred at the pubic symphysis and extending along bilateral adductor muscles, lateral to the greater trochanter, and along the iliacus muscle within the pelvis (Figure 2). No drainable collection was noted. Combined imaging findings of CT and MRI scans suggested emphysematous cellulitis or fasciitis. There was no drainable collection or surgical intervention, and the clinical signs and symptoms were not consistent with necrotizing fasciitis.

Gynaecology was referred to rule out pelvic inflammatory disease. However, they concluded that no active intervention was needed. Hepatobiliary advised on conservation management regarding liver laceration. Following multidisciplinary discussions, the decision was made to admit the patient under the care of the general medical and infectious disease team. The patient was also started on a treatment dose of enoxaparin for the non-occlusive IVC thrombus, likely secondary to an infection-driven hypercoagulable state. Enoxaparin was later changed to apixaban on day 4 of admission after a consultation with the haematology team.

During admission, Infectious Diseases was consulted due to the atypical presentation and imaging findings, and they noted that the extensive intraosseous and soft tissue emphysema observed on imaging was very unusual and challenging to explain. Therefore, further review with the surgical team was advised. The plan was to continue with IV meropenem and IV clindamycin.

Subsequently, blood cultures sent during the first day of admission grew *K. pneumoniae *on day 3 of admission, which, on confirmatory testing by the reference laboratory, was identified as belonging to the hypervirulent ST23 lineage, capsular type K1, positive rmpa and rmpa 2 genes. Susceptibility testing showed sensitivity to ciprofloxacin, cotrimoxazole, cefotaxime, gentamicin, and ertapenem. The patient received multiple antibiotics during her hospital stay, with initial empirical therapy followed by targeted de-escalation (Table 1).

**Table 1:** Antibiotic summary Targeted de-escalation

Day of admission	Antibiotic	Route	Context
1	Meropenem + Clindamycin	IV	Empirical
3	Meropenem + Clindamycin	IV	Blood culture confirmed positive Klebsiella Pneumonia
4	Meropenem	IV	Sensitivity released
10	Ciprofloxacin	Oral	De-escalation and continued for 12 weeks

**Figure 3:**
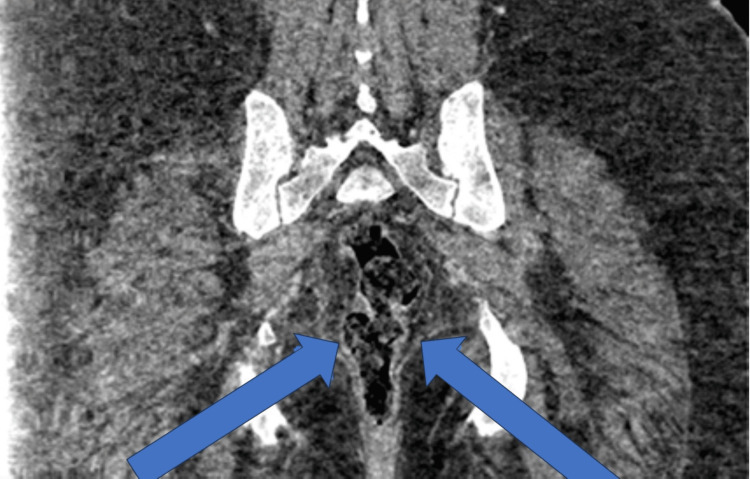
CT Neck showing the retropharyngeal phlegmon/abscess, Day 4 of admission Contrast‑enhanced CT revealing diffuse soft‑tissue swelling and retropharyngeal phlegmon extending into deep neck spaces without airway compromise

**Figure 4:**
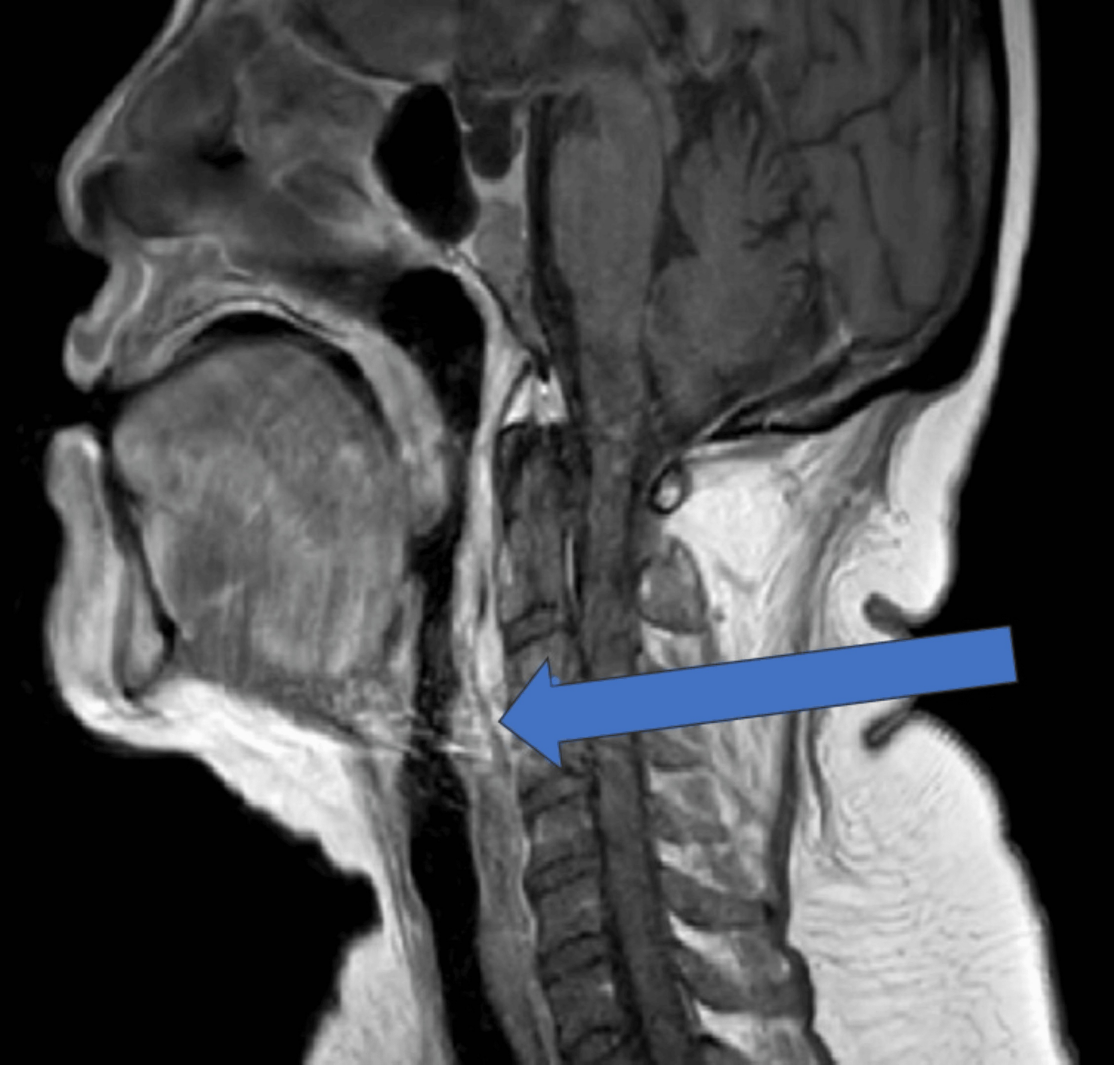
MRI of the neck showing shallow right retropharyngeal collection or abscess (improved), Day 7 of admission Improvement in retropharyngeal inflammatory changes with shallow residual collection, supporting response to conservative management.

On day 3 of admission, the Ear, Nose, and Throat (ENT) team was involved because the patient developed swelling on the right side of the neck and difficulty in swallowing. CT neck with contrast was performed, which showed marked diffuse soft tissue swelling involving supraglottic and infraglottic neck, including some mucosal space of the oropharynx, hypopharynx, retropharynx, and anterior soft tissue of the neck extending to the root of the neck, developing abscess/inflammatory phlegmon extending throughout the neck (Figure 3). ENT started the patient on regular IV dexamethasone. Three days after developing the swelling, it had reduced, and the patient reported that swallowing had improved. The subsequent MRI neck (Figure 4) after four days showed a shallow right retropharyngeal collection or abscess (improved). ENT decided that the patient should be conservatively managed with antibiotics, since there was no airway compromise and the abscess or inflammatory phlegmon was shallow. No culture sample was sent.

Patient was having hvKp that caused osteomyelitis and neck abscess. The patient improved clinically and radiologically, and on Day 24, she was discharged on apixaban for 12 months and oral ciprofloxacin for six weeks initially, which was extended to 12 weeks during clinic review.

During follow-up with the infectious disease team 73 days after admission, there was improvement of pelvic infection on the MRI scan, which was reported as significant resolution of the infective changes in relation to the anterior aspect of the pelvis. 

As shown in Table 2, the patient’s inflammatory markers and haematological parameters steadily improved during admission, with the white cell count and C-reactive protein normalising by day 61, reflecting a favourable clinical and microbiological response to treatment.

**Table 2:** Blood investigations trend C-reactive protein (CRP) and white blood cell count (WBC) progressively normalised by day 61, indicating treatment response. eGFR: estimated glomerular filtration rate

	Day 1	Day2	Day4	Day 9	Day15	Day21	Day32	Day61	Day 92	Reference value
White Cell Count	29.52	23.16	23.91	7.08	5.82	5.83	7.31	5.68	7.08	3.00-10.90x10^9
C- reactive protein	295	nil	175	41	112	89	23	13	4	0-5 (mg/L)
Sodium	131	135	132	130	133	132	138	137	138	133-146 (mmol/L)
Potassium	4.9	4.6	4.6	5.1	4.2	4.5	4.5	4.5	4.1	3.5-5.3 (mmol/L)
Urea	12.0	8.0	4.3	6.0	2.3	3.2	2.7	3.8	2.5	2.5-.7.8 (mmol/L)
eGFR	76	89	>90	>90	>90	>90	>90	>90	>90	>60 (ml/min)
Creatinine	72	63	47	42	38	43	49	44	44	49-90 (µmol/L)

## Discussion

This is a rare case in an elderly diabetic patient where a *K. pneumoniae* metastatic infection caused gas-forming osteomyelitis of the pelvic bones, a neck abscess, and a non-occlusive thrombus in the inferior vena cava. It also highlights the importance of a multidisciplinary and conservative approach in complex infections. This patient's first presentation was likely overlooked by the initial assessing clinician, as it was labelled as a “mechanical fall,” which could imply a fall was mainly due to an external, non-modifiable force [4]. A fall can be an initial signal of underlying illness or health decline, as “mechanical fall” patients often have recurrent falls and revisits to ED, as those with non-mechanical falls, and falls in the elderly need to be assessed thoroughly [1].

The World Falls Guidelines suggest performing a structured, multifactorial assessment to check for the risk of falls in elderly patients. These include mobility, medications, cognitive function, cardiovascular status, concerns about falling, vision, hearing, home hazards, pain, and nutritional status to create a risk profile and individualised management. We need to be proactive and opportunistic in taking history and examining the patient, as elderly patients often do not report when they have falls [5], as observed in our patient, who reported hip pain before the first fall, only on the second admission.

This patient’s recent travel history is a clue; hvKp used to be endemic to Asia. However, due to international travel and migration, hvKp has also been reported worldwide. hvKp strains possess virulence factors such as rmpA/rmpA2 for hypercapsule production, high-efficiency siderophores like aerobactin for iron scavenging, and specific K1/K2 capsular types, which together evade the immune system and disseminate to multiple organs [6]. Emphysematous osteomyelitis is an infrequent condition usually caused by gas-producing organisms like Enterobacteriaceae or anaerobes; however, Klebsiella species are being recognized as an emerging cause, particularly among diabetic patients [7]. HvKp infections are now also seen in immunocompetent individuals, with severe metastatic infections such as in the kidneys, lungs, and brain [8]. Pyogenic liver abscess is the classic presentation of hvKp infection. In our case, however, osteomyelitis and deep-seated abscesses were rare complications of disseminated hvKp infection, which can easily lead to misdiagnosis [9].

Multidisciplinary teams decided on a conservative treatment plan with close monitoring, which is the standard approach when there is no localised infection or spinal instability, as seen in another case report [10]. The management approach was in line with antimicrobial stewardship. Following initial broad-spectrum coverage with IV meropenem and IV clindamycin, antibiotic therapy was gradually de-escalated to oral ciprofloxacin for 12 weeks based on the organism's susceptibility. For systemic infections, meropenem with ceftriaxone has been reported to be effective as an empirical option. Treatment duration is usually six weeks, depending on the infection site and severity, as in this case, and is increased to a duration of 12 weeks as required [11].

This case has a different clinical manifestation from a recent case report of hvKp, which presented with classic manifestations such as liver abscess, gallbladder empyema, and infective endocarditis [12]. Instead, our patient had a more atypical course with emphysematous pelvic osteomyelitis, deep neck space infection, and IVC thrombus, in which the symptoms were initially masked by a presumed mechanical fall. While both cases highlight hvKp’s metastatic potential, our case demonstrates the diagnostic challenges when hvKp presents outside the typical hepatobiliary or cardioembolic spectrum, as illustrated by the atypical manifestations in our case report.

## Conclusions

Falls in elderly patients should be carefully assessed and not mislabelled as purely “mechanical”. Extra cautious assessment in elderly patients should be taken, as symptoms may be non-specific and easily labelled as mechanical falls or degenerative conditions, which can delay diagnosis, as observed in this case. This can also happen in young patients, for example, in leg pains for over two weeks. Clinicians should have a high suspicion of occult infections in patients with diabetes or immunocompromised patients. 

This case also illustrates a highly atypical and disseminated presentation of hvKp manifesting as emphysematous osteomyelitis, a deep neck abscess, and IVC thrombus. The initial attribution of the patient's symptoms to a "mechanical fall" underscores the diagnostic challenge posed by hvKp when it presents outside the classic hepatobiliary spectrum. Clinicians should maintain a high index of suspicion for occult disseminated infections, particularly in diabetic patients presenting with non-specific symptoms like falls or localized pain, and need to perform more advanced diagnostic tests. Due to hvKp strains becoming resistant, early recognition is crucial to prevent complications such as distal dissemination and an appropriate antibiotic coverage. A multidisciplinary approach is crucial for the management of such complex hvKp infections.
